# Food allergy

**DOI:** 10.1186/1710-1492-7-S1-S7

**Published:** 2011-11-10

**Authors:** Susan Waserman, Wade Watson

**Affiliations:** 1 Department of Medicine, Division of Clinical Immunology and Allergy, McMaster University, Hamilton, Ontario, Canada; 2 Department of Pediatrics, Dalhousie University, Division of Allergy, IWK Health Centre, Halifax, Nova Scotia, Canada

## Abstract

Food allergy is defined as an adverse immunologic response to a dietary protein. Food-related reactions are associated with a broad array of signs and symptoms that may involve many bodily systems including the skin, gastrointestinal and respiratory tracts, and cardiovascular system. Food allergy is a leading cause of anaphylaxis and, therefore, referral to an allergist for appropriate and timely diagnosis and treatment is imperative. Diagnosis involves a careful history and diagnostic tests, such as skin prick testing, serum-specific immunoglobulin E (IgE) testing and, if indicated, oral food challenges. Once the diagnosis of food allergy is confirmed, strict elimination of the offending food allergen from the diet is generally necessary. For patients with significant systemic symptoms, the treatment of choice is epinephrine administered by intramuscular injection into the lateral thigh. Although most children “outgrow” allergies to milk, egg, soy and wheat, allergies to peanut, tree nuts, fish and shellfish are often lifelong. This article provides an overview of the epidemiology, pathophysiology, diagnosis, management and prognosis of patients with food allergy.

## Introduction

In recent years, there has been a significant amount of media attention on the subject of food allergy. While the exact prevalence is unknown, recent estimates suggest that 6% of young children and up to 4% of adults in North America are affected by food allergy, and the prevalence of the disorder appears to be rising [1-4].

Food allergy is also a leading cause of anaphylaxis (a severe, potentially fatal allergic reaction; please see Anaphylaxis article in this supplement for more information) presenting to emergency departments [2]. Annually, there are approximately 200 deaths in the United States attributed to food allergy [5]. At present, there are no accurate data regarding food allergy-related deaths in Canada.

Accurate diagnosis and appropriate management of food allergy are critical since accidental exposure to even minute quantities of the food causing the allergic reaction may result in anaphylaxis [6]. This article provides an overview of current literature related to the epidemiology, pathophysiology, diagnosis, and appropriate management of food allergy. This review focuses primarily on immunoglobulin E (IgE)-mediated food-allergic reactions.

## Definition

The term food allergy is used to describe an adverse immunologic response to a food protein. It is important to distinguish food allergy from other non-immune-mediated adverse reactions to foods, particularly since more than 20% of adults and children alter their diets due to perceived food allergy [4]. Adverse reactions that are not classified as food allergy include food intolerances secondary to metabolic disorders (e.g., lactose intolerance), reactions to toxic contaminants (e.g. histamine produced by scombroid fish contaminated by Salmonella organisms) or pharmacologically active food components (e.g. caffeine in coffee causing jitteriness, tyramine in aged cheeses triggering migraine). Other conditions which are associated with symptoms similar to food allergy include auriculotemporal syndrome (a disorder characterized by facial flushing and salivation that may follow trauma to the parotid gland), and gustatory rhinitis [2-4].

## Pathophysiology

Although food allergy can arise to any food, the allergens responsible for more than 85% of food allergy are: milk, egg, peanut, tree nuts, shellfish, fish, wheat, sesame seed and soy [5]. These are also the “priority” allergens defined by Health Canada. It is the protein component, not the fat or carbohydrate component, of these foods that leads to sensitization and allergy. The allergenic segments or “epitopes” of these proteins tend to be small (10 to 70 kd in size), water-soluble glycoproteins that are generally resistant to denaturation by heat or acid and, therefore, can remain intact even after processing, storage, cooking and digestion [3,4,6]. Examples of these glycoproteins include caseins in milk, vicillins in peanut, and ovomucoid in egg. In general, allergies to additives and preservatives are uncommon.

Food-induced allergic disorders are broadly categorized into those mediated by immunoglobulin E (IgE) antibodies or by non-IgE-mediated mechanisms. IgE-mediated allergic responses are the most widely recognized form of food allergy and are characterized by the rapid onset of symptoms after ingestion. During initial “sensitization” to the food, consumption of the allergenic food protein stimulates production of IgE antibodies specific to that food which then bind to tissue basophils and mast cells. When the causal foods are subsequently eaten, they bind to their specific IgE antibodies and trigger the release of mediators, such as histamine, prostaglandins and leukotrienes, causing “clinical reactivity” and allergic symptoms. It is important to note that sensitization can be present *without* clinical reactivity, meaning that specific IgE to a food is present, but no reaction occurs with exposure [2-4,7,8]*.*

Non-IgE-mediated (cell-mediated) food allergy is less common and results from the generation of T cells that respond directly to the protein, leading to the release of mediators that direct certain inflammatory responses (e.g., eosinophilic inflammation) and can cause a variety of subacute and chronic disease states. These types of reactions typically affect the gastrointestinal (GI) tract and skin and include: dietary-protein-induced enterocolitis and proctitis, celiac disease and its related skin disorder dermatitis herpetiformis. Celiac disease is associated with a specific immunoglobulin A (IgA)-mediated sensitivity to gluten (a protein found in wheat, barley, rye and certain other grains), and is associated with chronic inflammation and damage to the villi of the small intestine. Dermatitis herpetiformis is a chronic skin disorder that may occur simultaneously with celiac disease or alone [2-4,7].

Some disorders are associated with a mixed IgE-/cell-mediated pathophysiology to food, such as atopic dermatitis, eosinophilic gastroenteritis and eosinophilic esophagitis (EoE; see articles on EoE and atopic dermatitis in this supplement). In these disorders, the association with food may not be demonstrated in all patients.

**Figure 1:**
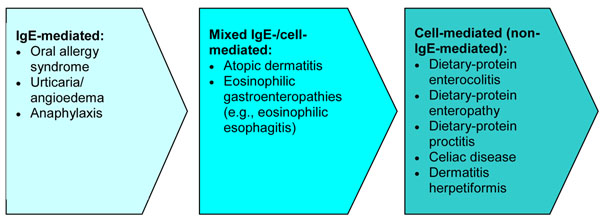
Spectrum of food allergy disorders according to pathophysiology [3,4,7]

The spectrum of food-allergy-associated disorders according to pathophysiology is shown in Figure 1. It is important to note that food allergy is not a cause of conditions such as migraines, behavioural or developmental disorders, arthritis, seizures or inflammatory bowel disease.

## Epidemiology

Many food allergies, particularly allergies to milk, egg, soy, and wheat, are usually outgrown within the first ten years of life [9]. In contrast, allergies to peanut, tree nuts, fish, and shellfish are often lifelong, although 20% of individuals may outgrow peanut allergy [10]. Peanut and tree nuts are responsible for the most serious allergic reactions and food-allergy related fatalities [11]. Canadian prevalence estimates for five of the major food allergens (i.e., peanut, tree nuts, fish, shellfish, and sesame) are shown in Table 1[14].

**Table 1:** Prevalence estimates for probable food allergy in Canada [14]

	Prevalence (%)	
**Food allergen**	**Children**	**Adults**

Peanut	1.68	0.71
Tree nuts	1.59	1.00
Fish	0.18	0.56
Shellfish	0.50	1.69
Sesame	0.23	0.05

Children with atopic disorders tend to have a higher prevalence of food allergy; approximately 35% of children with moderate-to-severe atopic dermatitis have IgE-mediated food allergy [12]. Studies have also shown that children with atopic dermatitis and food allergy have a higher prevalence of allergic rhinitis and asthma. Approximately 75% of children with atopic dermatitis develop allergic rhinitis and 80% develop asthma [13].

## Clinical manifestations

Food allergy is associated with a broad array of well-defined signs and symptoms, and can involve many body systems including the skin, GI and respiratory tracts, and cardiovascular system (see Table 2). Food allergy is not felt to play a role in chronic respiratory symptoms.

**Table 2:** Signs and symptoms of food allergy.

	IgE-mediated (immediate reactions)	Non-IgE-mediated (delayed/chronic reactions)
**Skin:**		
Urticaria	***√***	
Angioedema	***√***	
Erythema	***√***	***√***
Pruritus	***√***	***√***
Eczematous rash/lesions	***√***	***√***

**Respiratory:**		
Laryngeal edema	***√***	
Rhinorrhea	***√***	
Bronchospasm	***√***	
Nasal congestion	***√***	
Cough	***√***	
Chest tightness	***√***	
Wheezing	***√***	
Dyspnea	***√***	

**Gastrointestinal:**		
Angioedema of the lips, tongue, palate	***√***	
Oral pruritus	***√***	
Tongue swelling	***√***	
Vomiting	***√***	***√***
Diarrhea	***√***	***√***
Pain	***√***	***√***

**Cardiovascular:**		
Presyncope/syncope	***√***	
Hypotension	***√***	
Tachycardia	***√***	

Skin reactions are the most common clinical manifestations of allergic reactions to food and include acute urticaria (hives), angioedema (swelling) and erythema (redness of the skin). Typical respiratory tract symptoms include laryngeal edema, rhinorrhea, and bronchospasm. GI-related signs and symptoms of food allergy include nausea, vomiting, abdominal pain, and diarrhea.

The mildest IgE-mediated reaction is the oral allergy syndrome, which causes tingling and itching of the mouth and pharynx. This is typically triggered after consumption of fresh fruits and vegetables in pollen-allergic individuals. It is caused by cross reactivity of IgE antibodies to certain pollens with proteins in some fresh fruits and vegetables (see Table 3) [6]. For example, individuals with ragweed allergy may experience oropharyngeal symptoms following the ingestion of bananas or melons, and patients with birch pollen allergy may experience these symptoms following the ingestion of raw carrots, celery or apple. Fortunately, these proteins are heat labile, enabling allergic individuals to eat these foods when cooked. Allergy skin tests are usually negative to commercial food extracts in individuals with oral allergy syndrome, but are positive to the fresh food. Also, progression to systemic symptoms is rare, but may occur in a small proportion of patients with the condition [2-4,15].

**Table 3:** Oral allergy syndrome: cross reaction between proteins in pollen and fresh fruits and vegetables [6]

Pollen	Fresh fruit/vegetable/nuts	
Birch	• Almond • Apple • Apricot • Brazil nut • Carrot • Celery • Cherry • Coconut • Fennel • Hazelnut	• Kiwi • Nectarine • Peach • Peanut • Pear • Plum • Potato • Swede • Tomato • Walnut

Ragweed	• Banana • Cantaloupe • Cucumber	• Honeydew • Watermelon • Zucchini

Grass	• Cherry • Kiwi • Orange • Melon	• Peach • Potato • Tomato • Watermelon

The most severe reaction is anaphylaxis, which is defined as a serious allergic reaction that is rapid in onset and may cause death (please see Anaphylaxis article in this supplement for more information). The clinical criteria for diagnosing anaphylaxis are shown in Table 4[16,17]. There are numerous signs and symptoms of anaphylaxis which usually develop within minutes and up to 2 hours of food exposure. Early symptoms should not be ignored since reactions can be highly unpredictable, vary from person to person, and from attack to attack in the same person. This is especially true if there is a history of a previous anaphylactic reaction. Although peanut, tree nuts and shellfish are typically the most responsible culprits, anaphylaxis can be triggered by any of the common food allergens [6].

**Table 4:** Clinical criteria for diagnosing anaphylaxis [16,17]

Anaphylaxis is highly likely when any 1 of the following 3 criteria is fulfilled following exposure to an allergen:	
**1**	**Acute onset of an illness** (minutes to several hours) **with involvement of the skin, mucosal tissue, or both** (e.g., generalized hives, pruritus or flushing, swollen lips-tongue-uvula) **and at least 1 of the following:** a. **Respiratory compromise** (e.g. dyspnea, wheeze, bronchospasm, stridor, reduced PEF, hypoxemia) b. **Reduced BP** or associated symptoms of end-organ dysfunction (e.g. hypotonia [collapse], syncope, incontinence)

**2**	**2 or more of the following that occur rapidly after exposure to a *likely* allergen for that patient** (minutes to several hours): a. **Involvement of the skin-mucosal tissue** (e.g., generalized hives, itch-flush, swollen lips-tongue-uvula) b. **Respiratory compromise** (e.g., dyspnea, wheeze, bronchospasm, stridor, reduced PEF, hypoxemia) c. **Reduced BP** or associated symptoms (e.g., hypotonia [collapse], syncope, incontinence) d. **Persistent GI symptoms** (e.g., painful abdominal cramps, vomiting)

**3**	**Reduced BP after exposure to a* known *allergen for that patient** (minutes to several hours): a. **Infants and children:** low systolic BP (age specific) or > 30% decrease in systolic BP^*^ b. **Adults:** systolic BP < 90 mmHg or > 30% decrease from that person’s baseline

PEF = Peak expiratory flow; BP: blood pressure; GI: gastrointestinal

* Low systolic blood pressure for children is age specific and defined as: < 70 mmHg for age 1 month to 1 year; < 70mmHg + [2 x age] for age 1 to 10 years; < 90mmHg for age 11 to 17 years.

## Diagnosis

The diagnosis of a food allergy requires a detailed history and physical examination, and diagnostic tests, such as skin prick tests (SPT) and/or serum-specific IgE testing to foods (ImmunoCAP^®^). In some cases, oral food challenges may also be required [2-4].

Referral to an allergist is important to confirm the diagnosis of a suspected food allergy. Patients should avoid the food in question until assessment, and an epinephrine auto-injector should be prescribed, even if the diagnosis is uncertain [6].

### History

It is important to inquire about all suspect foods and to discuss the manner of food preparation (e.g. cooked, raw, added spices or other ingredients). Time of onset of symptoms in relation to food exposure, symptom duration and severity, as well as reproducibility of symptoms in the case of recurrent exposure should be determined. It is also important to ask about factors that can potentiate the allergic reaction, such as exercise or alcohol [2-4].

### Physical examination

The primary purpose of the physical examination is to look for supporting evidence of atopy and other allergic diseases (e.g., atopic dermatitis, asthma, and allergic rhinitis) and to rule out the presence of other conditions that may mimic food allergy. The physical examination is also useful for assessing overall nutritional status and growth in children.

### Diagnostic tests

In general, diagnostic tests for food allergy (e.g., SPT, serum-specific IgE tests, and oral food challenges) should be performed by an allergist. The SPT is a rapid, safe and sensitive method for diagnosing suspected IgE-mediated food allergy. A positive SPT appears as a wheal and flare reaction when the responsible food is applied to the skin and pricked. A positive SPT has a sensitivity of approximately 90%; however, its specificity is only around 50%. Therefore, a positive SPT alone is not sufficient for diagnosing food allergy; the patient must also have a supportive history. To minimize false positive results, over-testing with SPTs should be avoided. SPT should only be done for those foods that are relevant to the patient’s history. The negative predictive value of a SPT is greater than 95% and, therefore, a negative SPT generally confirms the absence of IgE-mediated reactions [2,15]. Although less sensitive and more costly than SPTs, serum-specific IgE tests can also be used for diagnosing food allergy, particularly if SPTs cannot be performed or are not available [4].

If there is still clinical suspicion of food allergy, but the diagnosis is uncertain based on the results of SPT and/or serum-specific IgE testing, than an oral food challenge may be appropriate. Oral food challenges involve gradual feeding of the suspected food with careful, medically-supervised assessment for any symptoms. In the event of symptoms, feeding is discontinued and the patient is treated where approriate. Food challenges should only be conducted in clinics or hospitals equipped with both the personnel and equipment needed to treat anaphylaxis [18].

Other strategies that can help assist in the diagnosis of food allergy are an elimination diet and food/symptom diaries. The elimination diet can be used for both the diagnosis and treatment of food allergy and requires complete avoidance of suspected foods or groups of foods for a given period of time (usually 1-2 weeks), while monitoring for an associated decrease in symptoms. Success of this approach in the diagnosis of food allergy depends on identifying the correct food allergen and completely eliminating it from the diet. It is limited by potential bias in both patients and physicians, and variable patient compliance with the diet. Food/symptom diaries require the patient to keep a chronological record of all foods eaten and any associated adverse symptoms. These records may be helpful for identifying the food implicated in an adverse reaction; however, they are not usually diagnostic, particularly when symptoms are delayed or infrequent [2-4].

Tests such as applied kinesiology, vega machine testing and serum immunoglobulin G (IgG) blood testing have no role in the diagnosis of food allergy. Again, if food allergy is suspected, the food should be avoided, an epinephrine auto-injector should be prescribed, and the patient should be referred for an allergy assessment.

## Treatment

There is currently no treatment for food allergy, beyond avoidance of the responsible food(s). Once a food allergy is diagnosed, strict elimination of the offending food allergen from the diet is necessary. A properly managed, well-balanced elimination diet can lead to resolution of symptoms while maintaining nutritional status. When the elimination diet is used as treatment, the identified food allergens are removed from the diet indefinitely, unless evidence exists that the food allergy has resolved [2-4].

In the case of accidental exposure, the treatment of choice is epinephrine administered by intramuscular injection into the lateral thigh [2-4]. There are currently two epinephrine auto-injectors available in North America: EpiPen^®^ and Twinject^®^. Both products come in two dosages (0.15 mg and 0.30 mg), which are prescribed according to weight. The 0.30 mg dosage should be used for those weighing 30 kg or more, and the 0.15 mg dosage for children weighing between 15–30 kg. Certain sources recommend switching to the 0.30 mg dose at 25 kg rather than 30 kg [19].

These devices should be stored properly (avoiding temperature extremes) and replaced before the expiration date. More information on these auto-injectors is available at http://www.epipen.ca and http://www.twinject.ca. All individuals receiving emergency epinephrine must be transported to hospital immediately (ideally by ambulance) for evaluation and observation [20].

A concise, written plan for the treatment of allergic reactions resulting from accidental exposure to the food should be developed, and copies made available to the appropriate persons (e.g., daycare providers, teachers, employers). Examples of such a plan, along with other relevant information and materials, can be downloaded at Anaphylaxis Canada (http://www.anaphylaxis.ca) or the Food Allergy and Anaphylaxis Network (http://www.foodallergy.org; a US-based association). Recommendations for the management of anaphylaxis in schools and other community settings [20] are also available through the Allergy Safe Communities website at http://www.allergysafecommunities.ca.

Patients and their caregivers must also be educated on food avoidance, the identification and treatment of allergic and anaphylactic reactions, the appropriate use of epinephrine auto-injectors, and how to obtain immediate medical assistance (e.g., call 911) (see Anaphylaxis article in this supplement for more information). Individuals should also be instructed to read food labels carefully, watching for hidden ingredients such as “natural flavour” or “spices” that may indicate the presence of allergens, as well as “may contain” warnings. All food-allergic patients should obtain and wear medical identification (such as a MedicAlert® bracelet/necklace) indicating their food allergy [6].

Recent research has also shown that peanut allergic children can be desensitized to peanut by feeding them increasing amounts of peanut under close supervision [21]. Similar studies have been conducted for egg and milk allergy. Results of these studies are promising; however, they have involved only small numbers of subjects, side effects have been noted, and protocols are still evolving. Therefore, further confirmatory studies in this area are needed.

**Figure 2:**
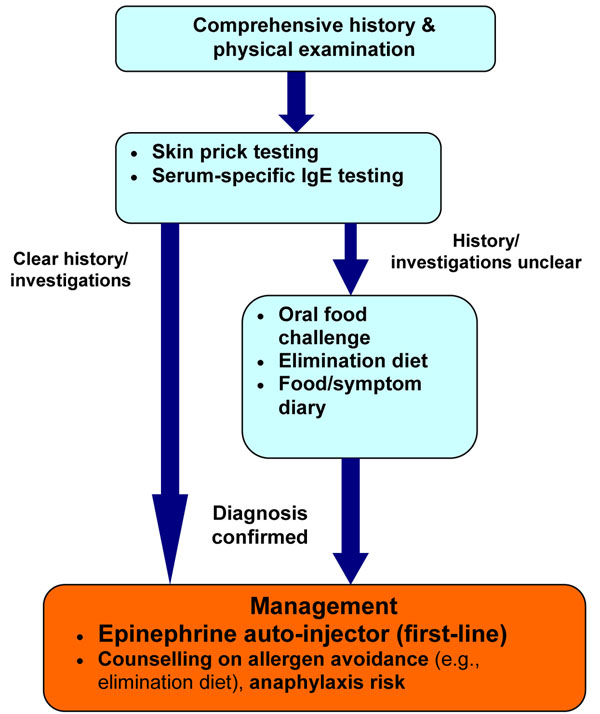
Simplified algorithm for the diagnosis and management of food allergy.

A simplified algorithm for the diagnosis and management of food allergy is provided in Figure 2.

## Prevention

Strategies for the prevention of food allergy have been extensively studied. Prior recommendations suggesting avoidance of highly allergenic foods in infant diets and the diets of pregnant/breastfeeding mothers have not been shown to decrease the prevalence of food allergy or atopic disease [6]. Recent guidelines from the American Academy of Pediatrics state that “no current convincing evidence exists to recommend specific avoidance of certain foods beyond 4-6 months of age for the prevention of allergy” [22].

## Prognosis

The prognosis of food allergy is complex and dependent on the particular food. As mentioned earlier, most infants and young children outgrow allergies to milk, egg, soy and wheat. Children should be re-evaluated at regular intervals to determine whether clinical tolerance has developed. The natural history of egg and milk allergy appears to be evolving in that an increasing number of children are outgrowing these allergies in their teenaged years [23,24]. Allergy to peanut, tree nuts, fish, and shellfish is more persistent, and lifelong in most cases.

## Conclusions

Food allergy is an important clinical problem of increasing prevalence. Assessment by an allergist is very important for appropriate diagnosis and treatment. Diagnosis currently relies on a careful history and diagnostic tests, such as SPT, serum-specific IgE testing (where appropriate) and, if indicated, oral food challenges. The mainstay of treatment is avoidance of the responsible food(s) and appropriate, prompt response to allergic reactions with epinephrine. Further insights into the pathophysiology of food allergy will lead to the development of improved methods for prevention, diagnosis, and management of the disorder.

### Key take-home messages

• Food allergy is defined as an adverse immunologic response to a dietary protein.

• Referral to an allergist is important for appropriate diagnosis and treatment.

• Diagnosis of a food allergy requires a detailed history, diagnostic tests such as skin prick tests (SPT) and/or serum-specific IgE testing to foods and, in some cases, oral food challenges.

• Treatment of food allergy involves avoidance of the responsible food(s) and injectable epinephrine.

• For patients with significant systemic symptoms, the treatment of choice is epinephrine administered by intramuscular injection into the lateral thigh.

• Most children "outgrow" allergies to milk, egg, soy and wheat by school age; allergy to peanut, tree nuts, fish, and shellfish are usually lifelong, although some patients may outgrow peanut allergy.

## Competing interests

Dr. Susan Waserman has received consulting fees and honoraria from AstraZeneca, GlaxoSmithKline, King Pharma, Merck, Novartis, Nycomed, and Paladin.

Dr. Wade Watson is a co-chief editor of *Allergy*, *Asthma & Clinical Immunology.* He has received consulting fees and honoraria for continuing education from AstraZeneca, GlaxoSmithKline, King Pharma, Merck Frosst, and Nycomed.
